# Ni/Ce_0.2_Zr_0.8_O_2_ Catalysts for Dry Reforming of Methane: Effects of Surfactant Amount on the Support Structure and Properties

**DOI:** 10.3390/ma18184329

**Published:** 2025-09-16

**Authors:** Haoran Sun, Xiaotian Zhou, Buhuan Wang, Tao Yang, Jingyi Yang, Ningyu Jia, Meng Zhang

**Affiliations:** 1 College of Chemistry, Zhengzhou University, Zhengzhou 450001, China; 2 School of Chemical Engineering, Zhengzhou University, Zhengzhou 450001, China; 3 Zhejiang Baima Lake Laboratory Company Limited, Hangzhou 310000, China

**Keywords:** dry reforming, Ni catalyst, CeZrO_2_, evaporation-induced self-assembly, surfactant

## Abstract

Dry reforming of methane (DRM) is an effective strategy to simultaneously convert CH_4_ and CO_2_ into valuable syngas. However, the widely employed Ni-based catalysts often suffer from rapid deactivation due to metal sintering and deposited carbon under harsh conditions. Herein, Ni/Ce_0.2_Zr_0.8_O_2_ catalysts were synthesized using the evaporation-induced self-assembly (EISA) method with the addition of the triblock copolymer surfactant P123. The addition of an appropriate amount of P123 improved the Ni dispersion; reduced Ni particle size; and enhanced the activation efficiency of both CH_4_ and CO_2_, thus increasing the reaction rate. In addition, the addition of P123 also enhanced the surface basicity and increased the concentration of oxygen vacancies of the catalyst, which enhanced its carbon removal capability and reduced deposited carbon. The catalyst with 0.2% P123 maintained excellent catalytic activity and stability for 300 min at 700 °C, with CH_4_ and CO_2_ conversion of 75% and 78%, respectively. These findings provide valuable guidance for the rational design of efficient and stable Ni-based catalysts for DRM.

## 1. Introduction

As is widely recognized, the extensive and continuous consumption of fossil fuels has led to a sharp increase in greenhouse gas emissions, mainly CH_4_ and CO_2_, which significantly accelerate global climate change [1,2,3]. Consequently, substantial research efforts have been devoted to converting these greenhouse gases into value-added chemicals, among which dry reforming of methane (DRM, CH_4_ + CO_2_ → 2CO + 2H_2_, ΔH_298K_ = +247 kJ·mol^−1^) has emerged as a particularly attractive strategy [4,5]. This process not only mitigates environmental pollution but also produces syngas with an H_2_/CO ratio close to unity, suitable for downstream applications such as Fischer–Tropsch synthesis and oxo-synthesis [6,7]. However, due to the highly endothermic nature of DRM and the chemical inertness of both CH_4_ and CO_2_ molecules, the reaction is typically operated above 700 °C. Although noble metal-based catalysts exhibit outstanding performance in DRM, their high cost limits large-scale application [8,9]. Alternatively, Ni-based catalysts exhibit comparable activity at a much lower cost, but they suffer from rapid deactivation due to sintering and carbon deposition under elevated temperatures [10,11].

The performance of Ni-based catalysts in dry reforming of methane (DRM) is widely recognized to depend on factors such as metal dispersion, metal–support interactions, and the ability to activate both CH_4_ and CO_2_ [12,13,14]. In particular, improving Ni dispersion can suppress carbon deposition, while supports with abundant basic and defect sites promote CO_2_ activation, thereby facilitating carbon removal and improving catalyst stability [15,16].

In recent years, ceria–zirconia solid solutions (CeZrO_2_) have attracted considerable attention as catalyst supports for DRM due to their high thermal stability, excellent redox properties, and adjustable surface basicity [17,18]. In an earlier study, we examined the effects of varied Ce/Zr ratios in the CeZrO_2_ supports prepared by the evaporation-induced self-assembly (EISA) method on the activity and stability of the Ni catalysts [19]. The results showed that the Ni/Ce_0.2_Zr_0.8_O_2_ catalyst with a Ce/Zr ratio of 1/4 exhibited the best performance. This was mainly attributed to the moderate Ce incorporation, which improved surface basicity, enhanced oxygen mobility, and strengthened the interaction between the metal and the support [20,21].

Based on these findings, further optimization of the catalyst structure is key to enhancing its catalytic performance. It should be noted that in EISA-based synthesis, surfactants act as soft templates that regulate precursor assembly and promote the formation of mesostructured frameworks. During synthesis, surfactant molecules interact with metal species and stabilize precursor species through electrostatic and coordination effects, thereby ensuring a more homogeneous distribution of the active phase [22,23]. Such interactions not only facilitate the formation of uniform pores and higher surface areas but also provide spatial confinement that effectively prevents Ni particle agglomeration during calcination and reduction [24,25]. In addition, these interactions further influence the evolution of the final pore structure [26,27]. Therefore, the type and amount of surfactant have a significant impact on the structural properties of the catalyst. They play a crucial role in mitigating sintering, enhancing Ni dispersion, and consequently improving the accessibility and stability of active sites for dry reforming of methane (DRM) [28,29].

Building on the previously established optimal support composition of Ce_0.2_Zr_0.8_O_2_, herein, we further investigated the effects of varying P123 surfactant amounts during support preparation on the structure, properties, and catalytic performance of supported Ni-based catalysts.

## 2. Experimental Section

### 2.1. Support Preparation

The Ce_0.2_Zr_0.8_O_2_ supports were synthesized via the EISA method using Ce(NO_3_)_3_·6H_2_O (purity 99.5%, Macklin Biochemical Co., Ltd., Shanghai, China) and ZrOCl_2_·8H_2_O (purity 98.0%, Macklin Biochemical Co., Ltd., Shanghai, China) as the precursors. In this work, the molar ratio of Ce/Zr was maintained at 1/4, while the amount of the triblock copolymer surfactant P123 (PEO-PPO-PEO, Mn~5800, Macklin Biochemical Co., Ltd., Shanghai, China) was systematically varied. Specifically, the molar ratios of P123 to the total metal ions were set at 0%, 0.2%, 0.4%, 0.6%, 0.8%, and 1%, respectively. Similar to the previous reports [30], the stoichiometric Ce(NO_3_)_3_·6H_2_O and ZrOCl_2_·8H_2_O were dissolved in ethanol (purity 99.7%, Ante Chemical Reagent Company, Suzhou, Anhui, China) to achieve a final metal ion concentration of 2 mol/L. The required amount of P123 was subsequently added to the solution. The mixture was sealed and stirred at room temperature for 10 h, then aged in an oven at 40 °C for 48 h. Subsequently, the gel was further heated at 100 °C for 24 h. The resulting solid was ground into a fine powder and calcined at 700 °C for 4 h in a muffle furnace (heating rate: 2 °C/min). Finally, the resultant supports were designated as 0C1Z4, 0.2C1Z4, 0.4C1Z4, 0.6C1Z4, 0.8C1Z4, and 1C1Z4.

### 2.2. Catalyst Preparation

Ni-loaded Ce_0.2_Zr_0.8_O_2_ catalysts were prepared by incipient wetness impregnation, with the nominal Ni content fixed at 5 wt%. Typically, the support was impregnated with an aqueous solution of Ni(NO_3_)_2_·6H_2_O (98%, Macklin Biochemical Co., Ltd., Macklin Biochemical Co., Ltd., Shanghai, China) at room temperature, then dried at 120 °C for 12 h and calcined at 700 °C for 4 h. The resulting catalysts were denoted as N*S*, where *S* denotes the corresponding support.

### 2.3. Catalyst Characterization

The catalysts were characterized by powder N_2_ adsorption, transmission electron microscopy (TEM), X-ray diffraction (XRD), H_2_ temperature-programmed reduction (H_2_-TPR), H_2_ chemisorption, H_2_-TPD, O_2_-TPD, CO_2_-TPD (Temperature-Programmed Desorption), diffuse reflectance infrared Fourier transform spectroscopy (DRIFTS), and thermogravimetric analysis (TGA). Detailed information is provided in the Supporting Information.

### 2.4. Catalyst Evaluation

The detailed information, including the evaluation process, gas chromatography information, typical gas chromatogram (Figure S1), and calculation methods, is described in the Supporting Information.

## 3. Results and Discussion

### 3.1. Physical Properties of the Catalysts

Figure S2a shows the N_2_-sorption isotherms of the as-prepared N*S* catalysts. All samples exhibit typical type IV isotherms with H1 hysteresis loops, indicating a characteristic mesoporous structure with cylindrical channels [31]. Notably, the isotherm of the N*S* catalyst with 0.2% surfactant is steeper and shows increased adsorption capacity, indicating a more uniform mesoporous structure upon surfactant addition [32]. The pore size distributions (Figure S2b), together with the specific surface area (SSA), pore volume (Vp), average pore size, and Ni dispersion in Table S1, further support this analysis. The N2 adsorption results indicate that the surfactant P123 does not fundamentally alter the pore structure, which is primarily dictated by the synthesis method. Nevertheless, 0.2% P123 notably enhances the surface area of the catalyst [33].

**Figure 1:**
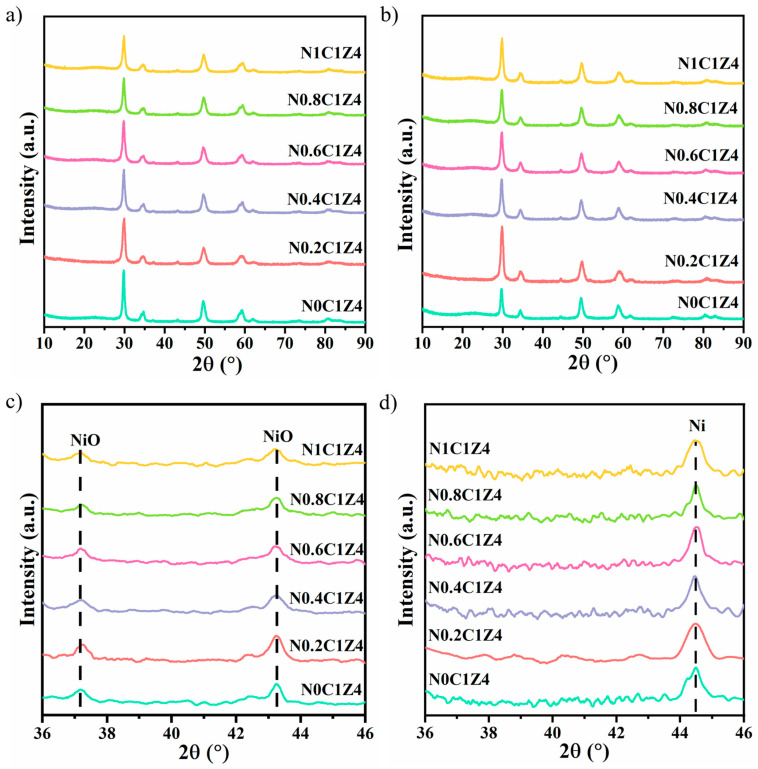
XRD patterns of the fresh (**a**) and reduced (**b**) N*S* catalysts; XRD patterns of the NxCZO catalyst NiO (**c**) before reduction and the active metal Ni (**d**) after reduction.

The XRD patterns of the fresh and reduced catalysts are shown in Figure 1a,b, respectively. In previous studies, the diffraction peaks of ZrO_2_ supports were typically assigned to the monoclinic (m) phase; however, upon Ce incorporation, the phase shifted to the tetragonal (t) structure [34,35]. This transformation is attributed to the insertion of Ce^4+^ ions into the ZrO_2_ lattice, since the ionic radius of Ce^4+^ is larger than that of Zr^4+^. It has been reported that CZO exhibits higher thermal stability [36,37]. Moreover, the addition of surfactant P123 does not affect the lattice parameters or crystalline structure of the catalysts [38]. This indicates that the surfactant primarily regulates the mesoporous structure while leaving the crystalline properties unaffected [39].

Figure 1c shows the partially enlarged XRD patterns of the fresh catalysts, while Figure 1d presents those of the reduced catalysts. As observed in Figure 1c, two characteristic diffraction peaks corresponding to NiO are clearly visible. Upon the addition of surfactant P123, the NiO peak intensity initially increases and subsequently decreases. A comparison of the peak widths of the N0C1Z4 and N0.2C1Z4 catalysts reveals significant broadening after surfactant addition. According to the Scherrer equation, broader peak widths correspond to smaller crystallite sizes. Calculations indicate that the catalyst without surfactant has an average NiO crystallite size of 18.34 nm, while the N0.2C1Z4 catalyst exhibits the smallest size of 12.79 nm. These results indicate that the addition of surfactant P123 during synthesis effectively reduces the size of the active metal particles and enhances their dispersion. Ni particles with smaller sizes expose more active surface sites, thereby increasing the conversion of CH_4_ and CO_2_ and enhancing the resistance to carbon deposition. These findings are in good agreement with the N_2_ adsorption–desorption results. The initial increase in NiO peak intensity may be attributed to the larger specific surface area induced by the surfactant, which facilitates greater loading of Ni species within the mesopores.

Figure 1d shows the magnified XRD patterns of the reduced catalysts. The characteristic NiO peaks disappear completely, being replaced by diffraction peaks of metallic Ni, indicating that the supported Ni species were fully reduced. The N0.2C1Z4 catalyst exhibits the highest diffraction intensity and broadest peaks, indicating the smallest Ni particle size and highest dispersion among all samples. However, further increases in surfactant content result in little change in Ni particle size. This suggests that while the surfactant influences the synthesis process, its impact on the final Ni particle size is limited.

**Figure 2:**
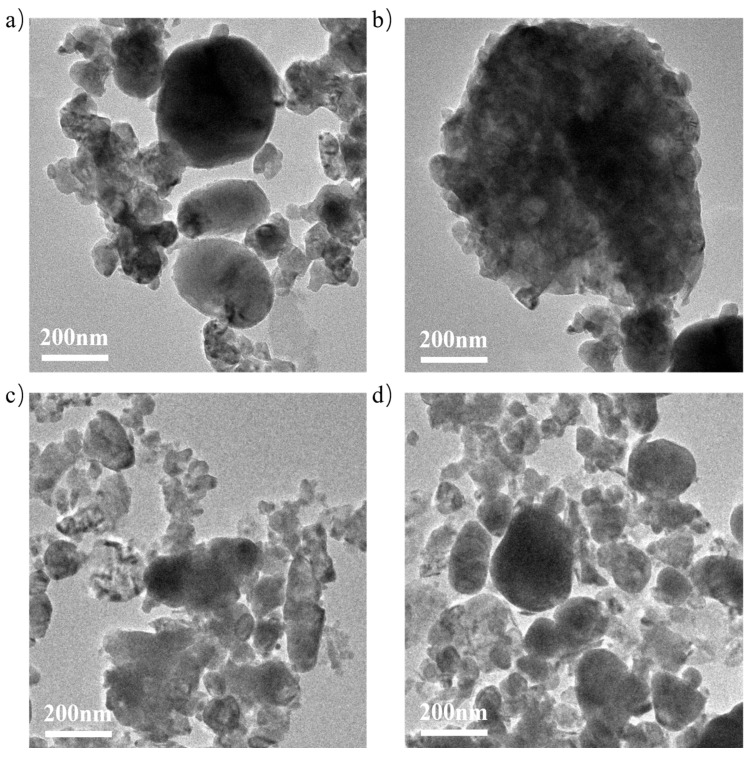
TEM images of the reduced catalysts ((**a**,**b**): N0C1Z4, (**c**,**d**): N0.2C1Z4).

Figure 2a–d present TEM images of the reduced NS catalysts. The addition of 0.2% P123 does not significantly alter the overall morphology but improves the dispersion of Ni particles on the surface, which is beneficial for catalytic activity in DRM. This observation is consistent with the XRD results. Figure S3 shows the HADDF images of the reduced NS catalysts and the corresponding elemental mappings. It can also be seen that the addition of 0.2% improved the dispersion of Ni particles on the catalyst surface.

### 3.2. Chemical Properties of the Catalysts

**Figure 3:**
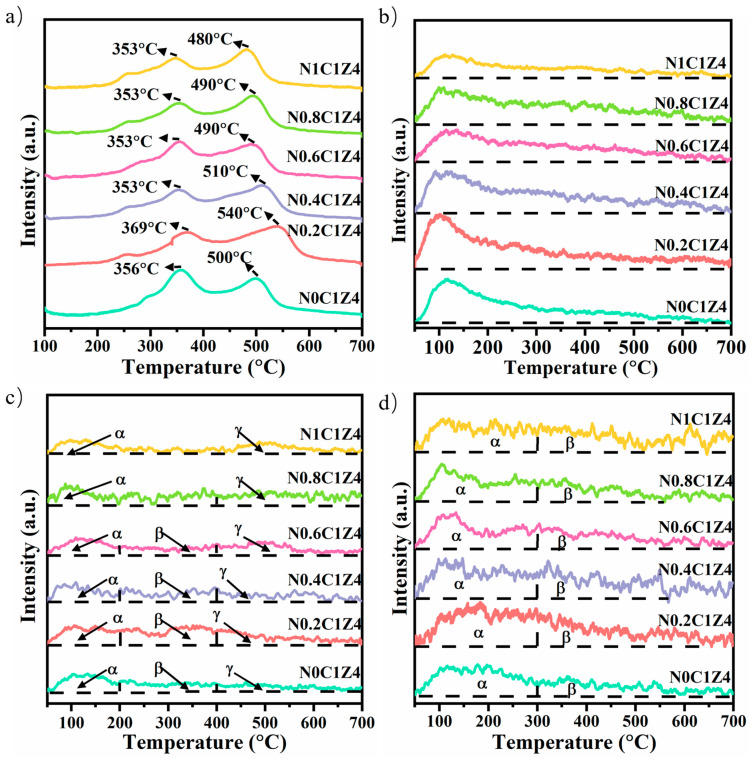
H_2_-TPR (**a**), H_2_-TPD (**b**), CO_2_-TPD (**c**) (α represents the weak alkaline sites, β represents the medium-strong alkaline sites, and γ represents the strong alkaline sites), and O_2_-TPD (**d**) (α represents the adsorbed oxygen species and β represents the lattice oxygen species) profiles of the N*S* catalysts.

Figure 3a shows the H_2_-TPR profiles of the employed N*S* catalysts. The hydrogen consumption peaks are closely related to the metal–support interaction (MSI). The reduction peak below 500 °C is attributed to NiO species weakly interacting with the support, whereas the peak above 500 °C corresponds to NiO species with strong MSI [40,41,42]. As shown in Figure 3a, the catalyst synthesized without surfactant exhibits two distinct reduction peaks, which are respectively assigned to NiO species with weak and strong MSI. Notably, the peak corresponding to weak MSI is more intense than that of strong MSI, indicating that metallic Ni on the surface primarily exists in a weakly interacting state. With the introduction of the surfactant, the reduction peak associated with strong MSI shifts toward higher temperatures, while the position of the low-temperature peak remains nearly unchanged, although its area decreases. Simultaneously, the area of the high-temperature peak increases, and it becomes noticeably broader, suggesting a wider distribution of Ni particle sizes on the support [43,44]. However, further increases in surfactant content cause the high-temperature peak to shift back to lower temperatures, eventually even below that of the sample without surfactant. This implies that the promotional effect of the surfactant on MSI is limited. Although the overall peak reduction temperatures slightly decline after the addition of the surfactant, the low-temperature peak area consistently decreases, while the high-temperature peak area increases. This observation still reflects an enhancement in MSI due to the presence of the surfactant. It is conducive to enhancing the stability of the catalyst and inhibiting its sintering.

To further evaluate the dispersion of active Ni species on the supports prepared with varying surfactant amounts, H_2_-TPD measurements were performed, as depicted in Figure 3b. Previous studies have demonstrated that under similar metal loadings, the area of the hydrogen desorption peak is positively correlated with the dispersion of metallic Ni [45,46]. As illustrated in Figure 3b, the introduction of surfactant leads to an increase in the H_2_ desorption peak area, indicating improved Ni dispersion. However, as the surfactant content further increases, a noticeable reduction in the H_2_ desorption peak area occurs, suggesting decreased Ni dispersion. This implies that excessive use of surfactant may hinder the effective dispersion of active Ni species on the catalyst surface [47]. These findings are consistent with the trends observed in the H_2_-TPR results.

Figure 3c shows the CO_2_-TPD profiles of the catalysts, which reflect their surface basicity. As observed, the desorption signals can be categorized into three distinct regions: peaks below 200 °C (α), those between 200 and 400 °C (β), and those above 400 °C (γ), which are attributed to the desorption of CO_2_ from weak, medium, and strong basic sites, respectively [48,49]. A comparative analysis among the catalysts reveals negligible variation in the weak and strong basic sites across all samples. However, upon the introduction of surfactant, a significant enhancement in the desorption peaks associated with medium basic sites is evident. In addition, a moderate increase in the α-region (weak basic sites) is also observed, indicating that the surfactant markedly enhances the overall surface basicity.

Notably, the intensity of γ-region peaks remains nearly unchanged, beneficial in avoiding potential deactivation of active metal species due to excessive surface basicity. As the surfactant content continues to increase, a gradual decline in the desorption peaks attributed to medium basic sites is observed, suggesting that an excessive amount of surfactant may adversely impact the catalyst’s basicity. Thus, optimizing the content of surfactant is essential to achieve balanced surface properties during catalyst synthesis.

Figure 3d shows the O_2_-TPD profiles of the catalysts, used to assess the concentration of oxygen vacancies and the mobility of oxygen species on the NS catalysts. As shown, all samples exhibit two characteristic desorption peaks: the low-temperature peak in the range of 50–300 °C is assigned to the desorption of Oα species, while the high-temperature peak in the range of 300–700 °C corresponds to the desorption of Oβ species [50,51]. The former is attributed to adsorbed oxygen species associated with oxygen vacancies, while the latter relates to the desorption of active lattice oxygen from the catalyst surface. Upon surfactant addition, both the Oα and Oβ desorption peaks noticeably intensify, indicating enhanced formation of oxygen vacancies and improved oxygen mobility. It favors CO_2_ dissociation and the formation of oxygen species, thereby promoting carbon removal. It can be correlated with the increased specific surface area induced by the surfactant, which provides more surface-active sites and oxygen defects. However, with further increases in surfactant content, both Oα and Oβ peak intensities gradually decline, indicating that excessive surfactant may hinder oxygen species adsorption and activation on the catalyst surface. This underscores the necessity of optimizing the content of surfactant to maintain favorable oxygen-related properties.

**Figure 4:**
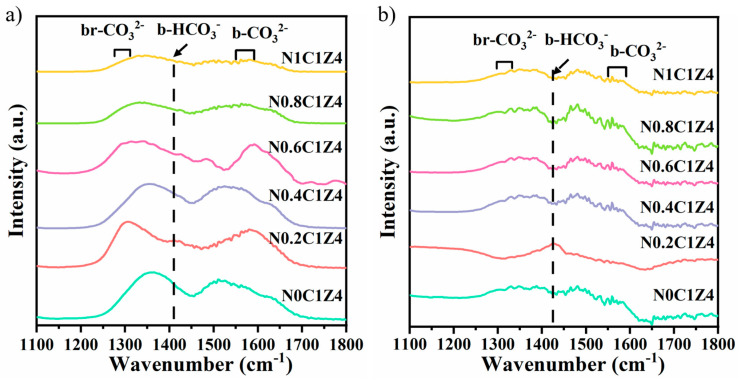
DRIFT spectra of CO_2_ adsorption on the N*S* catalysts at (**a**) room temperature and (**b**) 400 °C.

To further elucidate the intrinsic effect of surfactant addition on CO_2_ adsorption and activation, in situ DRIFT spectroscopy was conducted at room temperature and 400 °C, as shown in Figure 4. Vibrational bands corresponding to carbonate species formed from CO_2_ adsorption are observed in the 1100–1800 cm^−1^ range. Specifically, bands at ~1300, 1400, 1410, 1570, and 1630 cm^−1^ correspond to bridged carbonates, bidentate bicarbonates, monodentate bicarbonates, bidentate carbonates, and bridged carbonates, respectively. According to previous studies [52], these carbonate and bicarbonate species act as intermediates in the CO_2_ activation process, where the intensity of their vibrational bands correlates with the catalyst’s CO_2_ activation capacity. Notably, monodentate bicarbonates are considered more stable than other carbonate species and are less prone to decomposition during the reaction. In the 2200–2500 cm^−1^ region, the bands correspond to physically adsorbed CO_2_, with their intensity indicating the extent of CO_2_ adsorption on the catalyst surface [53]. Figure 4a shows that surfactant addition does not alter the types of carbonate species, which remain predominantly bidentate and monodentate, although monodentate bicarbonates appear more clearly. With further surfactant addition, the intensity of carbonate-related bands gradually declines, suggesting that excessive surfactant may hinder carbonate formation and CO_2_ activation. Beyond intensity variation, more subtle spectral differences are also observed. For instance, the spectrum of N0.8C1Z4 exhibits noticeable noise enhancement, as well as band shifts and partial splitting, implying possible structural heterogeneity or weaker adsorption stability at higher surfactant loading [54,55]. Figure 4b shows the DRIFT spectra at 400 °C. The CO_2_ adsorption behavior at high temperature is generally similar to that at room temperature, with all catalysts maintaining notable CO_2_ adsorption capacity. A key difference is the disappearance of the bridged carbonate band at elevated temperature, while the bands for bidentate and monodentate carbonates are significantly enhanced. Notably, monodentate bicarbonate species are only detected on the N0.2C1Z4 catalyst, exhibiting markedly higher intensity than in other samples. In contrast, the spectrum of N0C1Z4 becomes comparatively flattened at high temperature, reflecting weaker carbonate stabilization and a reduced ability to sustain CO_2_-derived intermediates [56,57]. Aside from this, the high-temperature spectra for the remaining catalysts show no distinct variation in band position or intensity, indicating that surfactant addition exerts limited influence on CO_2_ adsorption behavior under elevated temperatures [58,59].

### 3.3. Performance of the Catalysts

**Figure 5:**
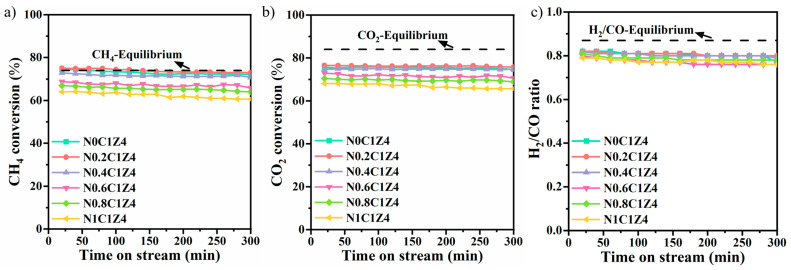
CH_4_ conversion (**a**), CO_2_ conversion (**b**), and H_2_/CO ratio (**c**) during DRM over the representative N*S* catalysts (reaction conditions: atmospheric pressure, WHSV = 24,000 mL·g^−1^·h^−1^, CH_4_/CO_2_ = 1/1, 700 °C).

As shown in Figure 5, the catalytic performance of the N*S* catalysts for DRM was evaluated at atmospheric pressure and 700 °C, with a WHSV of 24,000 mL·g^−1^·h^−1^. Although all the catalysts exhibited comparable stability, their catalytic activities varied significantly. Among them, N0.2C1Z4 showed the highest CH_4_ and CO_2_ conversions. Its initial CH_4_ conversion exceeded 75% and declined only slightly over time, attributed to abundant surface oxygen vacancies and well-dispersed active metal particles. Compared to the catalyst synthesized without surfactant, those prepared with surfactant demonstrated improved CH_4_ and CO_2_ conversions. However, excessive surfactant addition led to a decrease in catalytic activity. Based on the characterization results, this performance decline may be attributed to the weakened metal–support interaction caused by excessive surfactant coverage. Due to the reverse water–gas shift (RWGS) reaction, CO_2_ conversion exceeded CH_4_ conversion for all catalysts. Furthermore, the H_2_/CO ratio in the product gas remained below 1, also as a consequence of the RWGS reaction [60,61].

**Figure 6:**
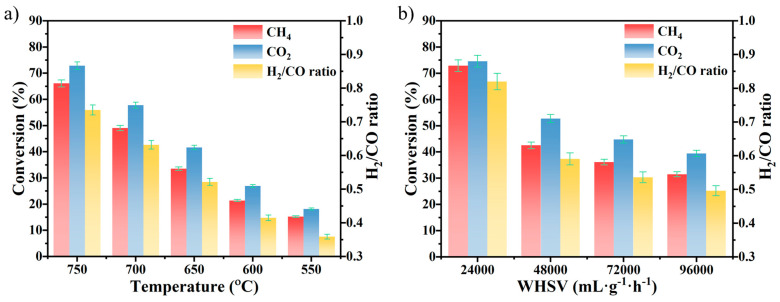
CH_4_ conversion, CO_2_ conversion, and H_2_/CO ratio during DRM over N0.2C1Z4 as a function of temperature (**a**) (reaction conditions: atmospheric pressure, WHSV = 24,000 mL·g^−1^·h^−1^, CH_4_/CO_2_ = 1/1) and WHSV (**b**) (reaction conditions: atmospheric pressure, CH_4_/CO_2_ = 1/1, 700 °C).

The effect of reaction temperature on N0.2C1Z4 catalytic activity was evaluated at atmospheric pressure, with a WHSV of 24,000 mL·g^−1^·h^−1^ and a CH_4_/CO_2_ ratio of 1:1. The results are shown in Figure 6a. Due to the endothermic nature of the DRM reaction, increasing the temperature significantly enhanced the conversions of both CH_4_ and CO_2_. Moreover, the concurrent occurrence of the reverse water–gas shift (RWGS) reaction resulted in consistently higher CO_2_ conversion compared to CH_4_, and the H_2_/CO molar ratio in the products remained below 1.0. With increasing temperature, the H_2_/CO ratio slightly increased, likely because DRM and CH_4_ cracking became dominant at high temperatures, occupying more active sites and suppressing the RWGS reaction. For reference, some representative catalytic performances of Ni-based catalysts supported on ZrO_2_, CeO_2_, and Ce–Zr solid solutions reported in the literature are summarized in Table S2, which further corroborates the trends observed in this work.

Figure 6b shows the DRM performance of the N0.2C1Z4 catalyst at atmospheric pressure and 700 °C under different WHSVs. The increase in WHSV leads to insufficient contact between the reactant gases and the catalyst, resulting in a gradual decrease in CH_4_ and CO_2_ conversions. In addition, as the contact time shortens, the H_2_/CO molar ratio also tends to decline, which can be attributed to the enhanced reverse water–gas shift (RWGS) reaction under higher CO_2_ concentrations [62].

### 3.4. Deactivation Analysis

Figure S4 shows the XRD patterns of the N*S* catalysts after being evaluated at 700 °C for 300 min, along with a partially enlarged view of the diffraction patterns. For catalysts prepared with surfactant, no significant changes in the support crystalline phase or Ni crystalline size were observed after the reaction. This suggests that surfactant addition enhances the thermal stability of the support and the sintering resistance of the catalyst. Additionally, compared with the reduced catalysts (Figure 1), the XRD patterns of the spent catalysts display characteristic diffraction peaks associated with deposited carbon, which is a major cause of catalyst deactivation [63].

**Figure 7:**
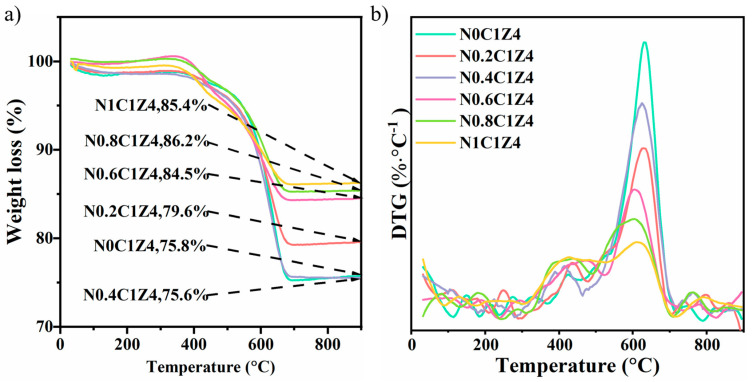
TG (**a**) and DTG (**b**) profiles of the N*S* catalysts after evaluated at 700 °C for 300 min.

To quantify deposited carbon, the spent catalysts were analyzed using thermogravimetric analysis (TGA) and derivative thermogravimetry (DTG). Among them, the catalyst with the highest deposited carbon is N0.4C1Z4, with a carbon content of 24.4%, while the catalyst with the lowest deposited carbon is N1C1Z4, with a carbon content of 14.6%. As shown in Figure 7, the weight loss peak at low temperatures (250–350 °C) corresponds to the oxidation of amorphous or activated carbon, whereas the high-temperature peak (>550 °C) is assigned to the oxidation of graphitic carbon. The temperature region between these two peaks is associated with the oxidation of filamentous carbon. The DTG profiles indicate that the carbon species formed on the catalyst surface are mainly inert filamentous carbon and graphitic carbon [64]. Among them, the amount of filamentous carbon formed on catalysts with different surfactant contents shows only slight variation, while the content of graphitic carbon differs significantly. The accumulation of graphitic carbon on the catalyst surface is considered the main reason for catalyst deactivation [65,66].

In addition, the catalyst without surfactant treatment exhibited the highest amounts of both filamentous and graphitic carbon. With increasing surfactant content, the amounts of both carbon types gradually decreased, and N1C1Z4 showed the lowest graphitic carbon content. TG data indicate that the total carbon on the catalyst with 0.4% surfactant is nearly the same as that on the catalyst without surfactant. However, the latter contains a higher fraction of graphitic carbon. These results indicate that the introduction of surfactant effectively reduces the graphitic carbon content on the catalyst surface, thereby improving its long-term stability. The relatively low graphitic carbon content in other catalysts may be due to their lower catalytic activity, resulting in reduced deposited carbon [67].

**Figure 8:**
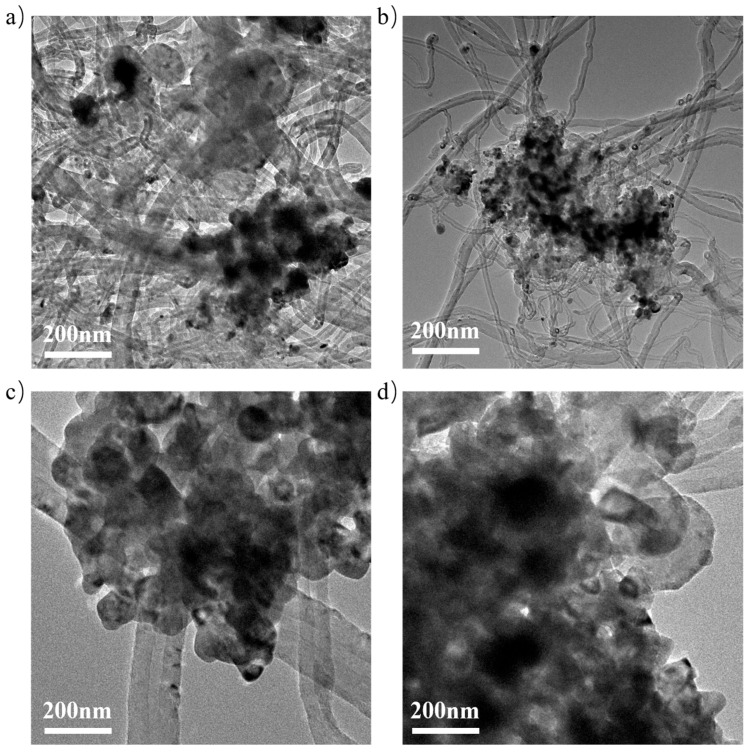
TEM images of the spent N*S* catalysts ((**a**,**b**): N0C1Z4, (**c**,**d**): N0.2C1Z4).

Figure 8 shows the TEM images of the N0C1Z4 and N0.2C1Z4 catalysts after evaluation for 300 min at 700 °C. It is evident that the amount of deposited carbon on the catalyst surface is significantly reduced with the addition of 0.2% surfactant, further confirming that the introduction of the surfactant enhances the catalyst’s resistance to deposited carbon. These findings are consistent with the TG results.

## 4. Conclusions

In this study, a series of Ce_0.2_Zr_0.8_O_2_ supports was synthesized via the EISA method with varying amounts of the surfactant P123. The performance of the corresponding Ni-supported catalysts for DRM was systematically investigated. The results demonstrated that the addition of an appropriate amount of surfactant significantly enhanced the specific surface area, oxygen vacancy concentration, and surface basicity of the catalysts. This, in turn, promoted the dispersion of active Ni species and improved both the activity and stability of the catalysts under DRM conditions. Among all the samples, the catalyst with 0.2% surfactant addition (N0.2C1Z4) exhibited the highest CH_4_ and CO_2_ conversions, superior resistance to sintering, and the lowest amount of graphitic deposited carbon. Characterization results indicated that moderate surfactant addition effectively optimized the metal–support interaction, enhanced oxygen mobility, and facilitated CO_2_ adsorption and activation. In contrast, excessive surfactant addition diminished catalytic performance by weakening metal–support interactions and reducing surface basicity. Moreover, TG analyses confirmed that the suppression of graphitic carbon formation played a vital role in maintaining the long-term stability of the catalyst. The relatively low content of graphitic carbon observed on catalysts with optimized surfactant content can be attributed to their improved oxygen vacancy concentration and enhanced catalytic activity.

In summary, precise regulation of surfactant amount during EISA synthesis provides an effective strategy to optimize the structure and surface properties of Ni/Ce_0.2_Zr_0.8_O_2_ catalysts, achieving high activity, robust stability, and excellent resistance to carbon deposition in DRM. These findings offer valuable guidance for the rational design of efficient and durable catalysts for DRM and other high-temperature reactions.

## Data Availability

The original contributions presented in this study are included in the article/Supplementary Material. Further inquiries can be directed to the corresponding author.

## Supplementary Materials

The following supporting information can be downloaded at: https://www.mdpi.com/article/10.3390/ma18184329/s1, Figure S1: A typical gas chromatogram during the DRM reaction; Table S1: Specific surface area, pore volume, average pore diameter and Ni dispersion of the NS catalysts; Table S2: A literature overview about the lifetime of the available Ni catalysts for DRM; Figure S2: N_2_-sorption isotherms (a) and BJH pore size distributions (b) of the N*S* catalysts; Figure S3: HADDF images and the corresponding elemental mappings of reduced N0C1Z4 (a) and N0.2C1Z4 (b); Figure S4: XRD patterns of the N*S* catalysts after evaluated at 700 °C for 300 min (a); XRD patterns of carbon deposition (b) and Ni (c) on the N*S* catalysts after evaluated at 700 °C for 300 min. References [68,69,70,71,72,73,74,75,76] are cited in the supplementary materials.
