# Supplementary material for: Ni/Ce0.2Zr0.8O2 Catalysts for Dry Reforming of Methane: Effects of Surfactant Amount on the Support Structure and Properties
Source: Materials (Basel). 2025 Sep 16;18(18):4329. doi: 10.3390/ma18184329 (PMC12471851; doi:10.3390/ma18184329)
Supplement: Supplementary file 1 [file materials-18-04329-s001.zip › materials-3813531-supplementary.pdf]

$Ni/Ce_{0.2}Zr_{0.8}O_2$ Catalysts for Dry Reforming of Methane: Effects of Surfactant Amount on the Support Structure and Properties

Haoran Sun [a], Xiaotian Zhou [b], Buhuan Wang [c], Tao Yang [a], Jingyi Yang [b], Ningyu Jia [a] and Meng Zhang [a]*

[a] College of Chemistry, Zhengzhou University, Zhengzhou, 450001, PR China

[b] School of Chemical Engineering, Zhengzhou University, Zhengzhou 450001, PR China

[c] Zhejiang Baima Lake Laboratory Company Limited, Hangzhou, 310000, PR China

*Correspondence: Corresponding author. College of Chemistry, Zhengzhou University, Zhengzhou, 450001, PR China. *Email*: zhangmeng1991@zzu.edu.cn.

Catalyst characterization

The Brunauer–Emmett–Teller (BET) surface area and Barrett–Joyner–Halenda (BJH) pore size distribution was measured using Micromeritics ASAP 2020 analyzer with N_2 adsorption at −196 °C. Prior to measurement, the samples were degassed under vacuum at 200 °C for 3 h.

Transmission electron microscopy (TEM) images were recorded on Tecnai G2 F20, which was equipped with an Energy-dispersive X-ray Spectrometer (EDX) detector. High angle annular dark field (HAADF) images were also achieved. Before each test, the sample was ultrasonically dispersed in ethanol for 30 min, and a drop of the suspension was placed onto a copper grid and dried at room temperature.

X-ray diffraction (XRD) patterns were recorded on SmartLab SE diffractometer with Cu Kα radiation source (λ = 1.5418 Å)(scan range: 10°-90°, step size: 0.02°, scan rate: 10 °/min, 40 kV, 15 mA).

H_2-TPR, H_2-TPD, H_2 pulse, O_2-TPD, and CO_2-TPD analyses were conducted using a Micromeritics AutoChem II 2920 instrument. For each test, approximately 200 mg of catalyst was loaded into a U-shaped quartz reactor and pretreated under flowing Ar (50 mL/min) by heating to 300 °C at 20 °C/min and holding for 60 min to remove surface impurities and adsorbed moisture.

For H_2-TPR, the sample was cooled to 100 °C under Ar, then exposed to 10% H_2/Ar (50 mL/min) and heated to 700 °C at 10 °C/min, with the H_2 consumption continuously monitored by a thermal conductivity detector (TCD).

For H_2-TPD, after the same reduction procedure as in H_2-TPR, the sample was cooled to 50 °C in He and purged for 20 min. After saturation with 10% H_2/Ar, He was used to remove weakly bound species, followed by heating to 700 °C at 10 °C/min while recording H_2 desorption via TCD.

For H_2 pulse chemisorption, after the same reduction procedure as in H_2-TPR, the sample was cooled to 50 °C in He and purged for 20 min. Then, H_2 was pulsed into the reactor to achieve the

saturated adsorption. Assuming that the adsorption stoichiometry (H/Ni) was 1, Ni dispersion was estimated.

$$Ni\ Dispersion = \frac{N_{surface}}{N_{total}}$$

$$N_{total} = N_{Ni} \times N_A$$

$$N_{Ni} = \frac{m_{Ni}(g)}{M_{Ni}(g/mol)}$$

where, $N_{surface}$ is the number of metal atoms on the surface, and the amount of hydrogen adsorption obtained from the hydrogen pulse experiment can be used to deduce $N_{surface}$. N_{total} is the total amount of Ni loading.

For O_2-TPD and CO_2-TPD, the reduced sample was cooled to 50 °C in He and purged for 20 min, then saturated with O_2 or CO_2. After purging with He, the sample was heated to 700 °C at 10 °C/min, and the desorption of O_2 or CO_2 was monitored by TCD.

In situ DRIFT spectra were recorded on Bruker Tensor 27 FTIR spectrometer (1000–4000 cm^{-1}, 4 cm^{-1} resolution, 64 scans per spectrum). Prior to each analysis, the sample was pretreated at 400 °C (10 °C/min) for 1 h in the *in situ* cell.

Thermogravimetric analysis (TGA) of the spent catalysts was performed on Netzsch STA 2500. The samples were heated from 30 °C to 900 °C at a rate of 10 °C/min under air, and the TG curves were recorded. The DTG profile was the first-order differential of the corresponding TG curve.

Evaluation

The DRM activity evaluation was performed on a micro quartz tube reactor with an internal diameter of 6 mm under the atmospheric pressure. For each test, the catalyst (200 mg, 20–40 meshes), diluted with the quartz sand (800 mg, 20–40 meshes), was placed at the center of the reactor. The sample was firstly reduced at 700 °C for 60 min in the flow of 10 vol % H_2/Ar (30 mL·min^{-1}). Then, the reactant gas was switched to a CH_4/CO_2 mixture (1/1, WHSV = 24,000 mL·g^{-1}·h^{-1}), and the effluent stream after cold-trap treatment was analyzed using an online gas chromatograph (PANNA A60)

equipped with a packed column (PANNA TDX-01, 3 m × 1.5 mm) and a TCD detector. The column oven temperature was set to 190 °C, and the bridge current was maintained at 50 mA. A typical gas chromatogram was shown in Fig. S1.

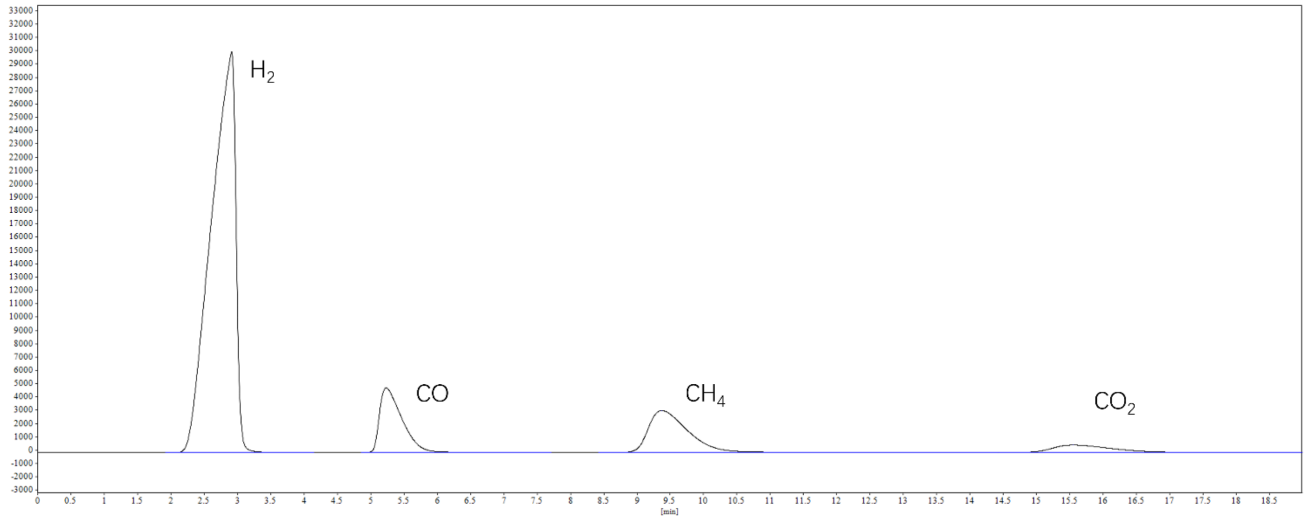

Figure S1 A typical gas chromatogram during the DRM reaction.

The conversions of CH_4 and CO_2, the H_2/CO ratio of the products, and the carbon balance were calculated using the following equations.

$$CH_4 \text{ conversion } (\%)=\frac{F_{in},CH_4-F_{out},CH_4}{F_{in},CH_4}\times 100\%, \quad (2\text{-}1)$$

$$CO_2 \text{ conversion } (\%)=\frac{F_{in},CO_2-F_{out},CO_2}{F_{in},CO_2}\times 100\%, \quad (2\text{-}2)$$

$$H_2/CO \text{ ratio}=\frac{F_{out},H_2}{F_{out},CO}\times 100\%, \quad (2\text{-}3)$$

where, $F_{in},X(X=CH_4 \text{ or } CO_2)$ were the flow rate ($mL\cdot h^{-1}$) of CH_4 and CO_2, respectively, at the inlet; $F_{out},X(X=CH_4, CO_2, H_2, CO)$ were the flow rate ($mL\cdot h^{-1}$) of CH_4, CO_2, H_2 and CO, respectively, at the outlet. The concentrations of all components were determined by external multi-point calibration based on peak areas. The flow rates of the feed and product gases were measured using a soap-film flow meter, and the carbon balance was maintained within 100 ± 5% to ensure the accuracy of the experiments.

Table S1. Specific surface area, pore volume, average pore diameter and Ni dispersion of the N*S* catalysts.

Sample	Specific surface area ($m^2 \cdot g^{-1}$)	Pore volume ($cm^3 \cdot g^{-1}$)	Average pore diameter (nm)	Ni dispersion (%) [a]
N0C1Z4	15.56	0.06	14.09	0.55
N0.2C1Z4	26.62	0.08	12.40	0.69
N0.4C1Z4	20.55	0.08	17.37	0.57
N0.6C1Z4	18.84	0.08	15.11	0.57
N0.8C1Z4	16.37	0.06	15.94	0.48
N1C1Z4	16.99	0.06	14.94	0.47

[a] Estimated by H_2 pulse chemisorption.

Table S2. A literature overview about the lifetime of the available Ni catalysts for DRM.

Catalyst	Reaction conditions	WHSV (mL/($g^{-1}\cdot h^{-1}$))	CH_4 conversion	CO_2 conversion	H_2/CO	Lifetime	References
$CeNi_{0.9}Zr_{0.01}Y_{0.09}O_3$	800 °C, $CH_4/CO_2/N_2$=3/3/1	42000	88%	91%	0.92	7 h	[1]
1GdRuCeZr	850 °C $CH_4/CO_2/N_2$=1/1/8	20000	51%	54%	0.9	50 h	[2]
Ru/CeZr	800 °C	50000	99.6%	/	1.9	200 h	[3]
Ni/ZrO_2-B	750 °C, CH_4/CO_2=1/1	24000	78%	71%	0.85	6 h	[4]
5Ni5YZr	700 °C	42000	52%	62%	/	7 h	[5]
$10Ni-Zn/ZrO_2$	800 °C, $CH_4/CO_2/N_2$=1/1/1	16100	89%	95%	1.8	100 h	[6]
$Ni-CeO_x/Al_2O_3$	700°C, $CH_4/CO_2/N_2$=1/1/1	120000	92%	92%	0.75	100 h	[7]
$5Ni/CeO_2$-G	700 °C, CH_4/CO_2=1/1	30000	62%	78%	0.7	50 h	[8]
$Ni@Co_1/CeO_2$	800 °C, $CH_4/CO_2/N_2$=1/1/1	90000	52%	68%	0.8	100 h	[9]

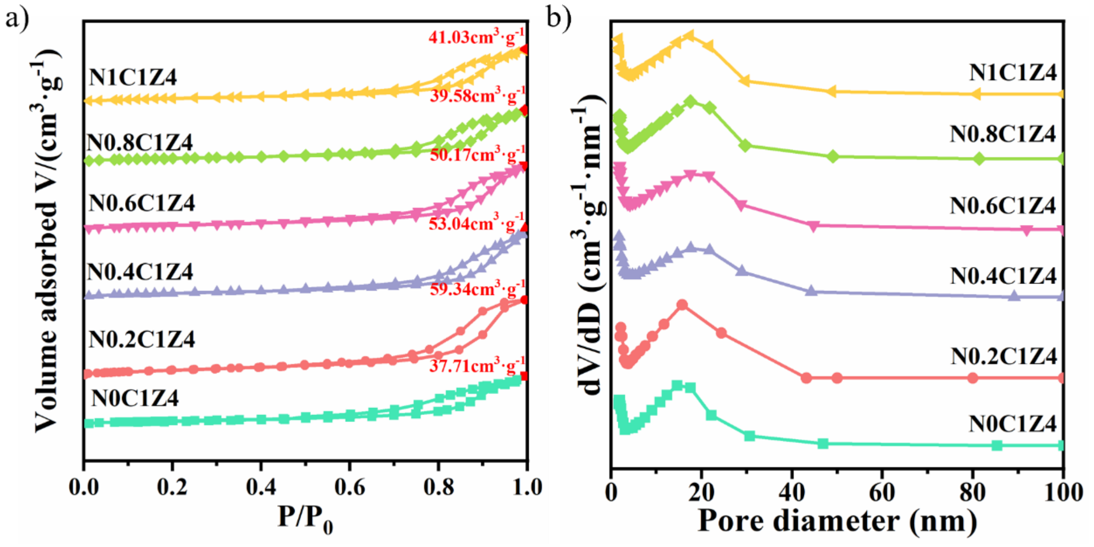

Figure S2 N_2-sorption isotherms a) and BJH pore size distributions b) of the N*S* catalysts.

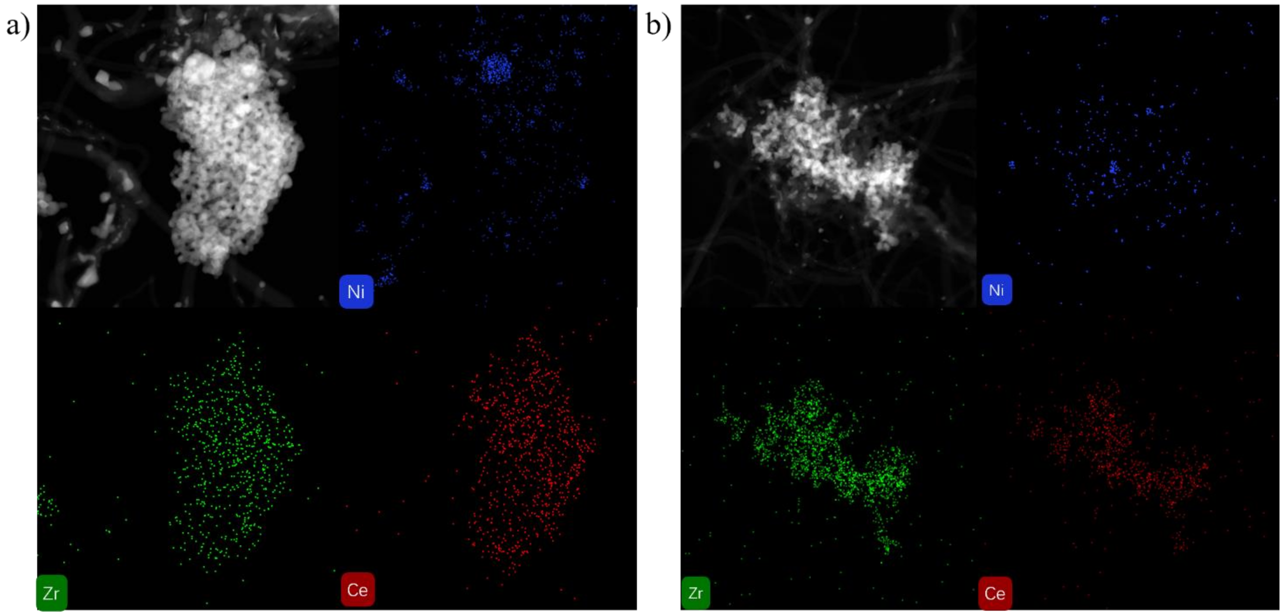

Figure S3 HADDF images and the corresponding elemental mappings of reduced N0C1Z4 a) and N0.2C1Z4 b).

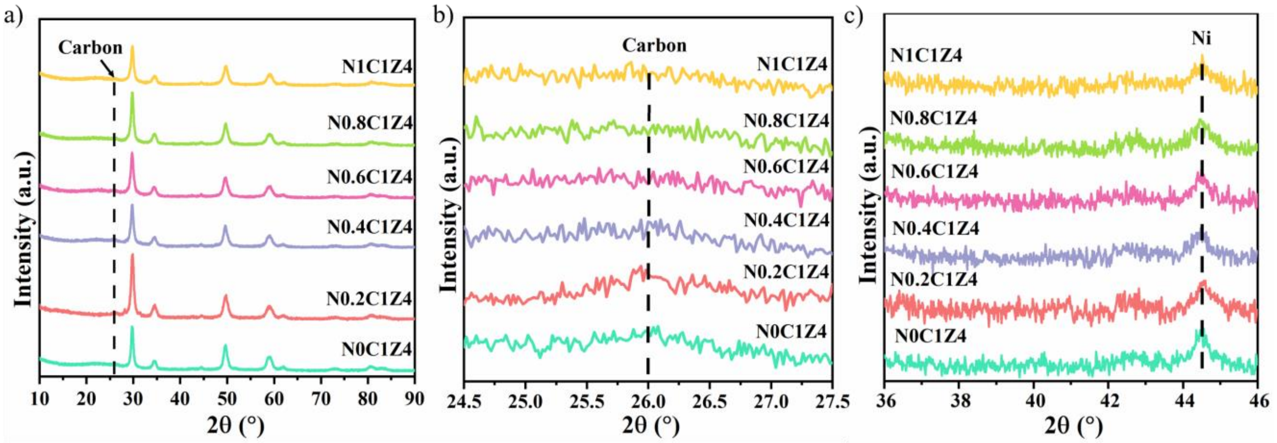

Figure S4 XRD patterns of the N*S* catalysts after evaluated at 700 °C for 300 min a); XRD patterns of carbon deposition b) and Ni c) on the N*S* catalysts after evaluated at 700 °C for 300 min.

References

[1] LANRE, M.S.; ABASAEED, A.E.; FAKEEHA, A.H. Modification of $CeNi_{0.9}Zr_{0.1}O_3$ Perovskite Catalyst by Partially Substituting Yttrium with Zirconia in Dry Reforming of Methane. *Materials* **2022**, 15(10), 3564.

[2] DAS, S.; SENGUPTA, M.; BAG, A. Gd–Ru Nanoparticles Supported on $Zr_{0.5}Ce_{0.5}O_2$ Nanorods for Dry Methane Reforming. *ACS Appl. Nano Mater.* **2021**, 4(3), 2547–2557.

[3] DAS, S.; GUPTA, R.; KUMAR, A. Facile Synthesis of Ruthenium Decorated $Zr_{0.5}Ce_{0.5}O_2$ Nanorods for Catalytic Partial Oxidation of Methane. *ACS Appl. Nano Mater.* **2018**, 1(6), 2953-2961.

[4] SHAO, J.; LI, C.; FEI, Z. MOFs-derived Ni@ZrO_2 catalyst for dry reforming of methane: Tunable metal-support interaction. *Mol. Catal.* **2024**, 558, 114028.

[5] FAKEEHA, A.H.; KURDI, A.; IBRAHIM, A.A. Performance Study of Methane Dry Reforming on Ni/ZrO_2 Catalyst. *Energies* **2022**, 15(10), 3841.

[6] VELISOJU, V.K.; VIRPURWALA, Q.J.S.; ATTADA, Y. Overcoming the kinetic and deactivation limitations of Ni catalyst by alloying it with Zn for the dry reforming of methane. *J. CO_2 Util.* **2023**, 75, 102573.

[7] WANG, S.; GUO, D.; HAN, M. Stabilizing Ni-CeO_x Bifunctional Nanoparticles on Activated Alumina to Enhance Carbon Resistance for Dry Reforming of Methane. *Ind. Eng. Chem. Res.* **2024**, 63(49), 21279-21289.

[8] XIA, H.; DANG, C.; ZHOU, D. Lamellar cross-linking Ni/CeO_2 as an efficient and durable catalyst for dry reforming of methane. *Chem. Eng. J.* **2024**, 489, 151365.

[9] YANG, E.; NAM, E.; JO, Y. Coke resistant NiCo/CeO_2 catalysts for dry reforming of methane derived from core@shell Ni@Co nanoparticles. *Appl. Catal. B: Environ.* **2023**, 339, 123152.
